# Alcohol consumption’s attributable disease burden and cost-effectiveness of targeted public health interventions: a systematic review of mathematical models

**DOI:** 10.1186/s12889-019-7771-4

**Published:** 2019-10-26

**Authors:** Ariel Esteban Bardach, Andrea Olga Alcaraz, Agustín Ciapponi, Osvaldo Ulises Garay, Andrés Pichón Riviere, Alfredo Palacios, Mariana Cremonte, Federico Augustovski

**Affiliations:** 1 Centre for Research in Epidemiology and Public Health, Institute for Clinical Effectiveness and Health Policy (IECS-CONICET), Dr Emilio Ravignani 2024 (C1014CPV), Buenos Aires, Argentina; 2 Institute for Clinical Effectiveness and Health Policy (IECS-CONICET), Dr Emilio Ravignani 2024 (C1014CPV), Buenos Aires, Argentina; 3 Center for Study of State and Society (CEDES), Sánchez de Bustamante 27 (C1173AAA) CABA, Buenos Aires, Argentina; 4 Group of Psychoactive Substances and injuries due to external cause, Institute of Basic, Applied Psychology and Technology (IPSIBAT) CONICET National University of Mar del Plata, Dean Funes 3250, B7602AYJ, Mar del Plata, Buenos Aires Argentina

**Keywords:** Alcohol, Burden of disease, Economic evaluations, Modelling

## Abstract

**Background:**

Around 6% of total deaths are related to alcohol consumption worldwide. Mathematical models are important tools to estimate disease burden and to assess the cost-effectiveness of interventions to address this burden.

**Methods:**

We carried out a systematic review on models, searching main health literature databases up to July 2017. Pairs of reviewers independently selected, extracted data and assessed the quality of the included studies. Discrepancies were resolved by consensus. We selected those models exploring: a) disease burden (main metrics being attributable deaths, disability-adjusted life years, quality-adjusted life years) or b) economic evaluations of health interventions or policies, based on models including the aforementioned outcomes. We grouped models into broad families according to their common central methodological approach.

**Results:**

Out of 4295 reports identified, 63 met our inclusion criteria and were categorized in three main model families that were described in detail: 1) State transition -i.e Markov- models, 2) Life Table-based models and 3) Attributable fraction-based models. Most studies pertained to the latter one (*n* = 29, 48.3%). A few miscellaneous models could not be framed into these families.

**Conclusions:**

Our findings can be useful for future researchers and decision makers planning to undertake alcohol-related disease burden or cost-effectiveness studies. We found several different families of models. Countries interested in adopting relevant public health measures may choose or adapt the one deemed most convenient, based on the availability of existing data at the local level, burden of work, and public health and economic outcomes of interest.

## Background

Alcohol is a cross-cutting, harmful factor considered in the United Nations’ Sustainable Development Goals (SDG) Agenda 2030, illustrating the issue that disease burden attributable to high consumption should be effectively addressed: SDG 3.5 aims to “strengthen prevention and treatment of substance abuse, including narcotic drug abuse and harmful use of alcohol” [1]. Alcohol consumption has been linked with more than 200 types of injuries and diseases, including road traffic and domestic accidents, cancer, liver cirrhosis, stroke, alcoholic cardiomyopathy and infectious diseases, and it has been estimated that around 6% of total deaths could be attributable to alcohol consumption worldwide [2]. The Institute for Health Metrics and Evaluation (IHME) estimated that in 2016 1.6% of total disability adjusted life years (DALYs) globally among females and 6.0% among males correspond to alcohol-related diseases [3]. A total 174,000 deaths were attributable to alcohol use disorders in that year in the Global Burden of Disease project [4]. About 12% of global DALYs due to road injuries and 14–18.5% of those due to self-harm and interpersonal violence are attributable to alcohol use. Alcohol is also the main risk factor for death and/or disability among people between 15 and 49 years of age in some regions of the world [3].

Alcohol consumption also generates an important economic burden [5]. Rehm and colleagues showed that its weighted average cost was 2.5% of GDP-purchasing power parity (PPP) among high-income countries (such as Canada, France, Scotland and United States); also that its average social cost of was 2.1% of GDP-PPP for some Asian middle-income countries (like the Republic of Korea and Thailand), and ranged between 0.45 and 5.44% of GDP in another study [6]. However, the discrepancies in the estimation methods and the cost components hinder direct comparisons, suggesting the need for local-level approaches. This economic toll will increase for LMICs because per capita alcohol consumption in these countries increases as GDP-PPP increases [7]. However, more precise estimates of the health, economic and social impact of alcohol are still lacking for many countries. Mathematical modelling is one of the principal methodologies to evaluate the potentiality of public health interventions through simulation. It has been increasingly used to evaluate the burden of disease of conditions and to estimate the cost-effectiveness of alternative healthcare interventions in order to efficiently allocate limited resources [8]. The Organization for Economic Cooperation and Development (OECD) has carried out an analysis based on a simulation model showing that several policies against high consumption of alcohol have also the potential to reduce regular and episodic drinking and alcohol dependence up to 5–10% [9]. Even the most expensive alcohol policies have shown favourable cost-effectiveness profiles. For example, brief physician advice (assuming 50% coverage), or raising taxes to alcohol have shown incremental cost-effectiveness ratios well-below one per capita GDP for many countries in all continents in a recent WHO report [10].

This would lead countries towards achieving the voluntary goal of reducing harmful alcohol consumption by 10% by 2025, adopted by the World Health Assembly in 2013 as part of the Global Noncommunicable Diseases (NCD) surveillance framework [11]. The ability of governments to design, implement and evaluate comprehensive prevention strategies, combining the strengths of different approaches, is critical to achieve this goal. In this regard, simulation models have many strengths [12]. They can synthesize available evidence and provide new evidence to inform decision making in areas where direct empirical research can be difficult or impossible, they can also extrapolate beyond data observed in primary research. However, they require multiple assumptions and depend on a variety of input parameters, especially epidemiological and economic ones, some of which may be of limited quality or even non-existent in many countries. That is why this study sought to establish which alcohol attributable disease burden and cost-effectiveness models have been tested in the world, based on a systematic review of the literature. Our objective was hence, to depict the main methodological characteristics of these models to inform researchers and technical teams in ministries of health their characteristics and pros and cons; thus, facilitating the choice of the model to use, adapt or develop in each country or region with the available data, in order to promote public policies aimed at reducing alcohol related problems.

## Methods

We followed the Meta-analysis Of Observational Studies in Epidemiology (MOOSE) [13] and Preferred reporting items for systematic reviews and meta-analyses (PRISMA) [14] statements to conduct and report the present systematic review. The present work is part of a larger multicentre study funded by the Ministry of Health of Argentina. The research question was to identify and evaluate those models to estimate population disease burden related to alcohol consumption, and cost-effectiveness of public health interventions aimed at reducing it. Due to the exploratory nature of this work a traditional PICO question format was not possible. We undertook a systematic search up to July 2017 in the following biomedical bibliographic databases: Medline, LILACS, ECONLIT, Psych Info, EMBASE and COCHRANE. Details about the electronic searches carried out are provided in the Additional file 1. We also hand-searched reference-lists of systematic reviews (SR) of models and health economic evaluations for additional information, and did prospective citation tracking.

### Selection process and eligibility

Pairs of reviewers independently selected articles initially by title and abstract and subsequently evaluated the full texts. Discrepancies were solved by consensus of the whole team. For the eligibility of articles the following inclusion criteria were established: 1) epidemiological models that explore alcohol related disease burden reporting attributable deaths and at least one of the following outcomes: DALYs, Quality-Adjusted Life Years (QALYs), or Years of Life Lost (YLLs), and 2) model-based economic evaluations of health interventions or policies, implemented or implementable at the city, state, or national level that included the aforementioned outcomes. In addition, studies fulfilling the previous criteria needed to be also comprehensive with regards to the attributable diseases or conditions included and cover at least three of these large areas: injuries by external causes, mental illness, gastrointestinal disease, cardiovascular or cancer.

The exclusion criteria were: 1) Publishing date before the year 2000, 2) Cost-only studies, 3) Model or study not specific to alcohol, or that assessed several effects and alcohol was not specified separately (e.g. considered several psychoactive substances), 4) only analysed subgroups (i.e. by age or sex), and/or did not refer to general population, 5) economic evaluations based only on randomized controlled trials (piggy back studies).

### Methodological quality evaluation and data extraction

Pairs of reviewers independently extracted data using a previously piloted data extraction form and assessed the risk of bias in the included studies using Cochrane’s Covidence software [15]. In case of disagreement, it was resolved by consensus. In case of difficulty to reach a consensus, a third author made the final decision. In order to evaluate the quality of each family of models the specific tool proposed by Bhuia et al. [16] was adapted. This tool involved an independent evaluation of the strengths (five domains) and limitations (five domains) of the ‘models’ based on their structure, specifications, assumptions, sensitivity analysis, validation, treatment of missing data, theoretical basis, incorporation of confounding factors and temporal window, and methodological limitations. Additionally, this tool was supplemented with a list of additional features agreed upon by the working group. Bhuia’s original tool is presented in the Additional file 1. We did not present the results of the risk of bias assessment at the individual study level because we aimed to assess and expose the methodological characteristics, strengths and limitations of the aggregate model conceptual families, with this very comprehensive tool.

Following Brennan et al. [12] we defined a model as a formal quantified comparison, synthesizing sources of evidence on costs and benefits, in order to identify the best option for decision makers to adopt. This author proposed a taxonomy of models [12] according to some dimensions that could be considered, such as cohort or individual level counts, and the allowance of interaction between individuals. We simplified this taxonomy grouping relevant articles into model *families* by means of their common central methodological approaches.

## Results

**Fig. 1:**
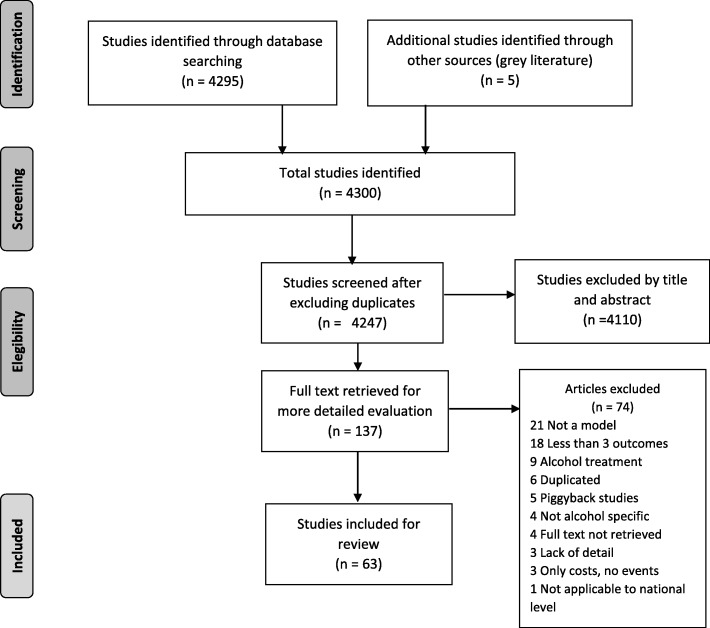
Flow of studies in the review

The flow of the systematic review is detailed in Fig. 1 (scheme according to the PRISMA checklist for systematic reviews).

We identified a total of 4295 references from the bibliographic databases and five more through grey literature, for further screening. Of them, 4247 remained after leaving out duplicates, and 137 were selected for full-text screening. We finally included sixty-three articles which were scrutinized in depth and grouped by their shared central methodological features, for reproducibility reasons. We identified a)state transition models, i.e., those decision-analytic modelling including Markov model cohort simulations and individual-based (first-order Monte Carlo) microsimulations [17], b) multistage life table models capable of estimating incidence, prevalence, remission, mortality based on population life tables, which predict the demographic consequences derived from introducing public health interventions [18], and c) population attributable and preventable fraction modelling studies, in which risk factors are selected based on the level of evidence for a causal relationship, relevancy of the risk factors for population health, availability and quality of population-representative data, and if the risk factors are avoidable [19]. Within these three broad families, comprising 70% of the publications identified in the systematic review (44 of 63), we describe more in depth the most well-known or utilized models, i.e. those with a larger number of citations, more extensively used, and with technical reports or papers describing their development and specifications. (See Table 1.)

**Table 1:** Main model families and their characteristics^a^

		State transition models (Markov)			Life tables models	Attributable fraction models	
	Model specifications	Sheffield	DYNAMO-HIA	Chronic disease model (CDM)	Life tables models	Comparative Risk Assessment	WHO-Choice
	Design main characteristics	Individual, no interactions, no history	Aggregate, no interactions, no history	Aggregate, no interactions, no history	Aggregate, no interactions, no history	Aggregate, no interactions, no history	Hybrid Population state transition/CRA model, aggregate, no interactions, no history
	Time Frame	Lifetime	10 years	Lifetime	Lifetime	Lifetime	Lifetime
	Main outcome	Disease Burden, cost-effectiveness	Mostly disease Burden	Disease Burden, cost-effectiveness	Mostly disease Burden	Mostly disease Burden	Disease Burden, cost-effectiveness
	Target interventions: tax / price (other than tax) / availability / advertising / short Screening (drinking and driving)	Several interventions evaluated	Alcohol price (tax)	Screening and brief interventions, and alcohol tax	Several interventions evaluated	Several interventions evaluated, mostly tax	Several interventions evaluated
	Key references	[20–31]	[32, 33]	[34, 35]	[36–39]	[40–67]	[68–71]
Objective	Clarity about the questions that the model aims to answer	Observed in all studies	Observed in all studies	Observed in all studies	Observed in all studies	Observed in all studies	Observed in all studies
	Description of the model structure (including formula)	Reported or referenced	Reported or referenced	Sometimes reported	Sometimes reported	Reported or referenced	Sometimes reported
	Model Specification	Yes	Yes	Yes	Yes	Yes	Yes
	Assumptions	Reported in all studies	Reported in all studies	Reported in all studies	Reported in all studies	Reported in all studies	Reported in all studies
Model family formulation and transparency	Derivations from the model (mentions if a specific dataset was developed)	Does not apply	Reported in all studies	Sometimes reported	Frequently reported	Does not apply	Sometimes reported
	Variables used in the model (response predictors, potential confounders)	Reported in all studies	Reported in all studies	Frequently reported	Frequently reported	Frequently reported	Reported in all studies
	Method of parameter estimation and inference	Reported in all studies	Reported in all studies	Frequently reported	Frequently reported	Frequently reported	Frequently reported
	Construction model process (Selection of variables)	Reported in all studies	Reported in all studies	Not available	Not available	Reported in all studies	Not available
	Diagnosis and testing of model adequacy	Not available	Not available	Not available	Not available	Not available	Not available
	Model Theoretical framework	Reported in all studies	Reported in all studies	Frequently reported	Frequently reported	Reported in all studies	Reported in all studies
Clear description of data	Data Sources	Reported in all studies	Reported in all studies	Reported in all studies	Frequently reported	Reported in all studies	Reported in all studies
	Data collection method	Reported in all studies	Reported in all studies	Frequently reported	Frequently reported	Reported in all studies	Frequently reported
	Process and size determination of the sample	Does not apply	Reported in all studies	Sometimes reported	Frequently reported	Does not apply	Does not apply
	Evaluation method (measurement data)	Reported in all studies	Reported in all studies	Reported in all studies	Reported in all studies	Reported in all studies	Reported in all studies
Model family findings: If information is available	Needed indicators (BoD measure / prevalence / incidence)	Reported in all studies	Reported in all studies	Reported in all studies	Frequently reported	Reported in all studies	Reported in all studies
	Standard errors and 95% confidence intervals	Not reported	Not reported	Reported in all studies	Frequently reported	Frequently reported	Reported in all studies
	Statistical adjustment model	Not reported	Not reported	Not reported	Not reported	Sometimes reported	Not reported
	Calibration with real data	Does not apply	Not reported	Not reported	Not reported	Not reported	Not reported
Validation	If model validation was undertaken	Frequently reported	Not reported	Not reported	Not reported	Not reported	Not reported
Sensitivity analysis	Sensitivity analysis was conducted	Reported in all studies	Not available	Reported in all studies	Frequently reported	Frequently reported	Reported in all studies
Missing data	If there is some explanation	Not available	Not available	Not available	Not available	Sometimes reported	Not available
Dissemination and involvement of experts	If the model is released before the end of the development or application of the model	Reported in all studies	Not available	Not available	Reported in all studies	Frequently reported	Reported in all studies
	If expert opinions were incorporated to develop the model	Reported in all studies	Not available	Not available	Not available	Frequently reported	Reported in all studies
Involvement of decision makers	Involvement of decision makers in the development model	Reported in all studies	Not available	Not available	Not available	Sometimes reported	Reported in all studies
	If a policy is recommended based on the results derived from the model	Reported in all studies	Reported in all studies	Frequently reported	Frequently reported	Frequently reported	Reported in all studies
Discussion of limitations	If possible methodological limitations of the model were discussed	Reported in all studies	Reported in all studies	Reported in all studies	Frequently reported	Frequently reported	Reported in all studies
	If the model is available and accessible to the user	Reported in all studies	Reported in all studies	Not available	Not available	Reported in all studies	Reported in all studies
Reproducibility	If data are available	Frequently reported	Reported in all studies	Not available	Not available	Sometimes reported	Reported in all studies
	If the codes are available	Not available	Not available	Not available	Not available	Not available	Not available
	If there is availability of a user manual model	Not available	Reported in all studies	Not available	Not available	Frequently reported	Reported in all studies
	Area of application	UK	Europe	National	National	Multiple areas	National
	Country/ies	Reported in all studies	Denmark, Finland, France, Germany, Ireland, Italy, Netherlands, Poland, Spain, Switzerland and Britain.	Holland	Australia	Reported in all studies	Various
	Sex	Reported in all studies (data by sex)	Reported in all studies (data by sex)	Reported in all studies (data by sex)	Not specified	Reported in all studies (data by sex)	Reported in all studies (data by sex)
	Age subgroups	Reported in all studies	Over 16 years. Categories every 10 years.	Reported in all studies population (no categories)	Reported in all studies population (no categories)	Reported in all studies	Reported in all studies
	Considers episodic risk consumption?	Yes	No	No	No	Yes	No
	Consider the context of consumption (detrimental level)?	Not reported	No	Not reported	Not reported	Frequently reported	Reported in all studies
	Categories used commonly consumed gr / day	Moderate (up to 21 units [10 ml of neat alcohol] per week for men and 14 units for women) /Hazardous (21–50 units men, and 14–35 women and Harmful (more than 50 units for men and more than 35 for women)	Alcohol consumption is measured by five categories of daily intake of grams of pure alcohol: 0–< 0.25 g/d, 0.25–< 20 g/d, 20–< 40 g/d, 40–< 60 g/d, ≥60 g/d.	for women: abstinence (no alcohol consumption), moderate (less than two standard drink units per day), excessive consumption (between two and four per day), and dangerous consumption (more than four per day), for men: abstinence (same), moderate consumption (fewer than four per day), excessive consumption (between four and six per day) and dangerous consumption (more than six per day)	Results in decrease in net grams or drinks (standard units) per day	Drinker I (females 0–19.99 g pure alcohol daily, males 0–39.99 g pure alcohol), Drinker II (females 20–39.99 g pure alcohol, males 40–59.99 g pure alcohol) and Drinker III (females ≥40 g pure alcohol, males ≥60 g pure alcohol).	Heavy or Dangerous (> 20 g per day for women and > 40 g per day for men)
	Consumption categories denomination	Moderate, hazardous and harmful	Not reported	Abstinence moderate, excessive and hazardous	Low, Harmful, Hazardous (WHO classification)	Drinkers I, II, III, (g/d). Sometimes differential category patterned	Alcohol consumption or dangerous heavy
	Reference category to calculate risks	Abstention	Abstention	Abstention	Abstention	Depends on the condition	Depends on the condition
	Incorporate elasticity (price, consumer demand)	Reported in all studies	Reported in all studies	Reported in all studies	Reported in all studies	Frequently reported	Reported in all studies
	Mortality from causes related health	Reported in all studies	Reported in all studies	Reported in all studies	Reported in all studies	Reported in all studies	Reported in all studies
	How many diseases or ICD codes incorporates? (number)	47	9	Not available	13	Variable	Variable
	Hospitalizations for related health conditions	Reported in all studies	Not reported	Sometimes reported	Reported in all studies	Frequently reported	Reported in all studies
	Includes damage to third parties (crime, AFS)	Reported in all studies	Not reported	Not reported	Reported in all studies	Sometimes reported	Not reported
	Incorporates indirect costs (labor productivity, absenteeism, unemployment)	Reported in all studies	Not reported	Not reported	Not reported	Not reported	Not reported
	Incorporates quality of life / disability (DALYs, QALYs)	Reported in all studies	Not available	Reported in all studies	Reported in all studies	Reported in all studies	Reported in all studies

^a^Some models did not fall into these broad categories and their main features are described in the text

Most studies pertained to the “Attributable fraction-based models” family (*n* = 29, 46.0%), followed by state transition one (*n* = 16, 25.4%) and the life-table family (*n* = 4, 6.3%). Some of the studies run at the individual-level (microsimulations) while others assessed aggregate populations or cohorts. None explored the interaction of either individuals, or multiple individual behaviours or risk factors. None of them was a discrete event simulation.

Briefly they encompass the following:

*State transition models* include three main specific models: the Sheffield Alcohol Policy Model, the Dynamic Modelling for Health Impact Assessment (Dynamo-HIA) model and the Chronic Diseases Model. Transitions in disease-related states were more commonly modelled than risk-factor-related behaviors transitions (for example between various levels and patterns of alcohol use). The Sheffield Alcohol Policy Model (SAPM) provides estimates of the effectiveness and cost-effectiveness of policies to reduce alcohol consumption. It has been used in Great Britain, Canada and multiple European countries to examine the potential impact of pricing and promotion policies; minimum unit prices and restrictions on discounts; regulation of alcohol output density and licensing hours; advertising controls; alcohol screening and brief intervention strategies. It is one of the most complete and complex models we identified. It includes different sub-models: those specific to the intervention, and the main simulation model of expected risk consumption, which is a Markov-type chain that models the annual risk of death for each alcohol-related illness. The results of the model provide estimates of: changes in alcohol consumption for different subgroups of the population; changes in the incidence of various alcohol-related injuries, including health conditions, crime, unemployment and absence due to illness; direct costs to health services or to the police, as well as costs associated with changes in the quality of life of people (for example, of alcohol-related illnesses or being a victim of a crime). It allows analysing particular subgroups of interest, such as dangerous young drinkers, moderate low-income drinkers or high-income women who drink alcohol. There are many publications that describe the experience with the use of SAPM [20–31, 72].

The DYNAMO-HIA model can be used to quantify the impact of changes in risk factors in multiple diseases, comparing a reference scenario with one or more intervention scenarios. It is a Markov type mathematical model that allows to simulate the expected events in a population from different risk factors distribution scenarios. The model requires data on: 1) the population (size, births, survival, weight of the DALYs); 2) incidence, prevalence, relative risks and mortality of the different conditions that it evaluates; 3) prevalence of risk factors and risk categories for the assessed risk factor, in this case alcohol. As results of the model, information is obtained on the number of deaths, disease-free life expectancy, number of cases of cardiovascular disease, stroke, diabetes, lung cancers, oral cavity, oesophageal, colorectal and breast, and COPD. It has been used in countries of the European Union to estimate the impact of different scenarios of price increase through taxes.

Lhachimi et al. [32, 33] published a *dynamic modelling tool,* named DYNAMO-HIA-A for generic health impact evaluations, used for alcohol. The generic model meets three technical criteria (real population, dynamic projection, explicit risk factors states) and three usability criteria (modest data requirements, results of enriched models, generally accessible).

Tariq et al. in 2009 [73] published a cost-effectiveness analysis to assess the effects of a screening and brief intervention (SBI) for excessive alcohol consumption (compared to the current state of not doing so) in the primary care setting, in The Netherlands, using the Chronic Disease Model. It incorporated the perspective of health. It is able to establish the costs of medical care and the cost for QALYs earned after the intervention. It is a non-specific mathematical model for alcohol. The model provides a simulation of a real-life population. It also incorporates the effectiveness of brief interventions. However, the specific group of diseases attributable to alcohol included was unclear. Van den Berg in 2008 [35] had already used this model, but for Sweden, and instead of SBIs, it incorporated the increase in alcohol taxes, determining that it results in a cost-effective intervention in that country.

Regarding *life table models*, Cobiac et al. [72] published a study in which they evaluated the cost-effectiveness of eight interventions to reduce the damage attributable to alcohol and determine the optimal intervention combination. These included taxes, advertising bans, increasing the legal minimum drinking age, licensing controls for hours of operation, brief intervention (with and without general medical telemarketing and support), drinking driving campaigns, random testing and residential treatment for alcohol dependence (with and without naltrexone). This study was conducted for the population of Australia in 2003. Health outcomes were assessed using DALYs, a multi-cohort and multi-state approach based on life tables to determine changes in disease incidence, prevalence and mortality, and alcohol-related injuries due to each intervention. It is specially designed to cope relatively easily with a large number of diseases simultaneously, allowing also for comorbidities [74]. Byrnes et al. in 2010 also used the life-table model to estimate the benefits of tax increases on alcoholic beverages in Australia [72].

As regards *attributable fractions models*, comparative risk analyses (CRA) were developed by the WHO working group, among others. This method allows estimating and comparing the burden of disease and mortality attributable to different risk factors. In the case of alcohol, the exposure (prevalence) of different forms of consumption (average and episodic heavy drinking) is considered, all the health conditions related to alcohol, its prevalences’, and the relative risks of these forms are identified. With these data, the attributable population fractions are estimated, generally according to sex, age groups and regions, comparing the current exposure with the counterfactual exposure (usually lifetime abstention). These analyses estimate the burden of disease attributable to alcohol in a region, commonly in the form of DALYs.

The WHO-Choice Model has been called Generalized Cost-Effectiveness Analysis (GCEA) and is based on the WHO Choosing Cost Effective Interventions (WHO CHOICE) project. Methodologically, it can also be considered a continuation of the Comparative Risk Assessment but it also uses state transitions. In the GCEA, the cost-effectiveness of certain interventions (or their combinations) is evaluated through a population status transition model (POPMOD). It compares scenarios in which different interventions are implemented that run for 10 years, and others without them (natural history). These scenarios are usually projected over a 100-year horizon. The data required for the model are: prevalence of risk exposure, associated morbidity and mortality rates, relative risks, remission rates, effect sizes of each intervention to be modelled, and costs of implementing each intervention for 10 years. The effectiveness of each scenario is frequently measured in DALYS, and the cost-effectiveness by the cost per DALY avoided.

Finally, we identified a number of miscellaneous models, not fitting in the above-described groups. Chikritzhs et al. in 2002 [75] for example, described a model for calculating estimates of the number of deaths caused by alcohol. They describe a common approach for both acute and chronic conditions related to alcohol. In the absence of consistent measurements of the prevalence of risky alcohol consumption from national surveys, they recommend the use of per capita consumption data in order to adjust the etiological fractions of the population consuming alcohol over time and between regions. The parameters evaluated were mainly of sensitivity and specificity of screening, and effectiveness of brief interventions. Solberg et al. in 2008 [76] published a systematic review and economic evaluation based on models they denominate “algebraic model”. The main parameters evaluated were adherence, effectiveness, lifetime burden of diseases attributable to alcohol; and costs. Navarro et al. published in 2011 a cost-effectiveness study on brief interventions to prevent the misuse of alcohol in rural areas of Australia. They compared nine detection scenarios with the current state. Its parameters are related to consumption and Alcohol Use Disorders Identification Test (AUDIT) questionnaire levels based on the analysis of a survey in 1540 subjects in rural Australia. Doran et al. in 2013 [77] examined the economic and health implications of changing alcohol taxes in Australia through a model-based economic evaluation that combines tax aspects with epidemiological modelling, for multiple alcohol cohorts, using parameters such as taxes, consumption and elasticity. Lewsey et al. in 2016 [78] developed an alcohol policy model that calculates life years, quality adjusted life years (QALYs), and health care costs using AUDIT and other risk factors’ screening. Its parameters are based on Scottish surveys and morbidity records. Finally, Chung et al. in 2014 [79] explored the effects of age, period and cohort on alcohol-related mortality in relation to changes in government taxes on alcohol. Its parameters were retrospective mortality data of more than 30 years between 1981 and 2010 in Hong Kong. Alcohol-related mortality was evaluated from chronic causes, acute causes, all causes (chronic and acute) and causes 100% attributable to alcohol.

The central features of these families of models of disease burden or cost-effectiveness, and their methodological quality appraisal are presented in the table.

## Discussion

Through this systematic review we identified 63 studies that described and reported relevant results using a variety of models to assess the burden of disease and / or the cost-effectiveness of interventions for alcohol control. These models included comparative risk assessments, life table models, and state transition ones, as broad categories.

Our results show that a variety of alcohol consumption specific modelling approaches have been used to understand its associated burden. This observation is in agreement with a recent review of modelling structures for interventions on alcohol and other drugs’ dependence, published by Hoang et al., and using Brennan’s taxonomy [80]. Also coincident with our findings, the comprehensive OECD report, which summarized the results of the economic evaluations of alcohol policies available in 2015, retrieved most analyses found in our study [9]. However, our review incorporates many descriptive elements regarding their reporting and conduction quality, through the modified Bhuia tool to assess economic evaluations’ quality. We observed that some of the evidence is based on the WHO-CHOICE model developed by the World Health Organization (WHO) in the early 2000s. In addition, the Australian ACE prevention program (corresponding to the family “life tables models” in our study), the Chronic Disease Model (related to the “comparative risk assessment”), the SAPM (“state transition models”) and Dynamo-HIA were used in many countries. We incorporated a miscellaneous category of important models which did not constitute conceptual and application units, clearly separated from the rest [9]. Some of the models like WHO-CHOICE, Dynamo, SAPM and CDM developed special software packages or webpages, where end users can enter parameters for different settings or countries and get results. Many of these evaluations characterized the effect of price increases -mainly through taxing-, advertising restrictions, and prohibitions as the three most cost-effective policies to reduce alcohol-related harm. Short interventions within the health system, have also shown to be efficient, but costlier than population strategies, and for some policies such as school programs and media campaigns, effectiveness is not fully established.

The SAPM model allows to have a wide spectrum of consequences attributable to excessive alcohol consumption since it contemplates, not only direct damage to health, but also the social consequences of consumption, including accidents and injuries, as well as impact on the economy of individuals and the society (indirect costs). It was designed and adapted to evaluate the impact and cost-effectiveness of multiple interventions to control alcohol consumption so it’s very versatile. However, it requires a great deal of information with a high level of discrimination that can be very difficult, even impossible, to obtain in many countries. DYNAMO-HIA has a user-friendly graphic interface, and employs a model structure that ensures accurate simulation using epidemiological evidence while having modest data needs. It allows sensitivity analyses, although not probabilistic ones. It is available for free download and includes a data set covering a large number of countries. However, results do not account for broad effects that a change in alcohol consumption may have on global population health. CDM has been used for projections of alcohol consumption and disease prevalence, estimates of health-adjusted life expectancy and cost-effectiveness analysis but not to capture societal costs and consequences. It has a limited range of attributable diseases. Also, long term effectiveness of interventions, needed as a model parameter might be difficult to ascertain. Life table models are based on widely available data in most countries (mortality and population life tables), and by incorporating incidence, prevalence, and other natural history data, can assess the consequences of different public health interventions, usually estimating burden (in DALYs) and healthcare system costs. They are somewhat less complex than other model families and less “data hungry”, though are less suitable or easily adaptable if focusing on non-health outcomes, or wider societal costs. Comparative risk assessments’ (CRA) main advantage is that it allows to model and compare multiple diseases and risk factors simultaneously. When used to model alcohol burden of disease or interventions to address it, it considers the damage from different levels of exposure including volume, pattern, or even context of drinking. On the other side, it is unable to model interactions, and it might require important amounts of data, that may not be available for all regions or countries. Finally, the WHO CHOICE model is part of a larger and well-known project that was used to estimate disease burden and cost-effectiveness of multiple conditions and interventions in different regions of the world, and therefore an advantage is that much of the information required by the model may be readily available. As with other “generic” models, it is not properly designed to measure non-health outcomes, so it has a limitation to capture certain societal costs and effects falling outside the boundaries of the health system.

Similar reviews of modelling of cost-effectiveness or disease burden have been done in various areas of public health. For example, in smoking cessation [81], weight management interventions [82] and tuberculosis screening [83], state-transition Markov models were coincidently the most frequently used method. A population-scale simulation modelling approach can provide a solid basis to evaluate the relative effectiveness and cost-effectiveness of a range of alcohol prevention and control strategies, overcoming the limitations of other approaches, and providing constant estimates of inputs from resources, costs and results. A case-based microsimulation approach offers the best option for modelling realistic individual life trajectories, considering the heterogeneity in populations and individual behaviours that can influence alcohol harm, as well as the relative effectiveness of the policies in population groups. These models capture the complex set of interrelations between the previous and current use of alcohol, its demand, and the health, social and economic consequences it causes.

A limitation of the present work may be the simplification of the classification of modelling approaches into “big” families, which may not reflect the real choices countries could count on, based on the model aspects and the real-world complexity. Also, other aspects of economic evaluations such as perspectives of costs and benefits, or discounting, were not evaluated. Despite these limitations, we have provided a very comprehensive picture of the state-of-the-art in public health alcohol modelling. We have critically appraised studies with respect to the appropriateness and quality of the modelling aspects.

The significant burden of disease attributable to alcohol consumption, and the associated economic and social damage, warrants the debate on possible public policies with the aim of preventing and reducing problematic consumption of alcoholic beverages. Among these measures, the increase in the prices of alcoholic beverages through taxes with the intention of reducing their affordability is one of the most cost-effective interventions [10, 84–87].

Decision tree modelling may not be sufficient for evaluating treatments for alcohol, and we did not identify this type of model fulfilling our inclusion criteria. The state transition modelling could be sophisticated enough to capture many of the potential developments in a disease process through the use of a series of health states. It can accommodate the time dimension, as individuals move over time in different states and also depict the heterogeneity of cohorts by allocating them into relevant (although limited) groups. However, it relies on many simplifications, and -especially in the case of cohort state transition models- cannot add past events and personal attributes in determining transition probabilities, nor allow participants to transit to the next state at different time intervals. Individual based models represent a powerful tool as it is possible to simulate multiple events and incorporate subjects’ “histories”, but they require a larger number of parameters and may be computationally intensive. When evidence is needed to move forward alcohol public health decisions, which may demand substantial budget allocation, a complex model would be preferable in case several complex strategies are being considered. The choice of the most adequate modelling approach, time frame and perspective of costs and benefits useful for a country may change the modelling results and policy implications significantly. Countries should carefully consider the availability of existing data at the local level, as well as the type of interventions desired to be implemented. Future evaluations should continue to identify the variety of techniques for modelling public health interventions to counter alcohol hazardous consumption.

## Conclusions

Our systematic review of alcohol attributable disease burden and cost-effectiveness models shows extensive literature exists on the subject. We categorized the relevant models identified into three main families according to their core methodological aspects -state transition models (both individual level and cohort-based); life-table models; and attributable fraction models-. All include some more complex and “data hungry” specific structures, as well as some less complex and data-intensive ones. We incorporated many descriptive elements of their quality of reporting and conduction. The summary information reported in our paper can be helpful for researchers and decision makers planning to undertake this type of studies, in the light of local data availability and interventions desired to be tested.

## Supplementary information

**Additional file 1: Appendix 1.** Search Strategy. **Appendix 2.** Checklist for model evaluation, and score frame.

## Data Availability

All data generated or analyzed during this study are included in this published article and its supplementary information files.

## Abbreviations

(AUDIT): Alcohol Use Disorders Identification Test
(CDM): Chronic disease model
(CRA): Comparative risk assessment
(DALYs): Disability adjusted life years
(DISMOD-MR): Disease Modeling – Metaregression
(Dynamo-HIA): Dynamic Modelling for Health Impact Assessment
(GBD): Global Burden of Disease
(GCEA): Generalized Cost-Effectiveness Analysis
(IHME): Institute for Health Metrics and Evaluation
(MOOSE): Meta-analysis Of Observational Studies in Epidemiology
(NCD): Noncommunicable Diseases
(OECD): Organization for Economic Cooperation and Development
(POPMOD): Population status transition model
(PPP): Purchasing power parity
(PRISMA): Preferred reporting items for systematic reviews and meta-analyses
(QALYs): Quality-Adjusted Life Years
(SAPM): The Sheffield Alcohol Policy Model
(SBI): Screening and brief intervention
(SDG): United Nations’ Sustainable Development Goals
(WHO CHOICE): WHO Choosing Cost Effective Interventions project
(WHO): World Health Organization
