# Supplementary material for: Alcohol consumption’s attributable disease burden and cost-effectiveness of targeted public health interventions: a systematic review of mathematical models
Source: BMC Public Health. 2019 Oct 26;19:1378. doi: 10.1186/s12889-019-7771-4 (PMC6815367; doi:10.1186/s12889-019-7771-4)
Supplement: Supplementary file 1 — Additional file 1: Appendix 1. Search Strategy. Appendix 2. Checklist for model evaluation, and score frame. [file 12889_2019_7771_MOESM1_ESM.docx]

# Appendix 1. Search Strategy

**PubMed (NLM) 22-06-2017**

| Search | Query |
| --- | --- |
| [#27](https://www.ncbi.nlm.nih.gov/pubmed/advanced) | Search **(#20 AND #26)** |
| [#26](https://www.ncbi.nlm.nih.gov/pubmed/advanced) | Search **(#21 OR #22 OR #23 OR #24 OR #25)** |
| [#25](https://www.ncbi.nlm.nih.gov/pubmed/advanced) | Search **Drinker*[tiab]** |
| [#24](https://www.ncbi.nlm.nih.gov/pubmed/advanced) | Search **Alcoholic*[tiab]** |
| [#23](https://www.ncbi.nlm.nih.gov/pubmed/advanced) | Search **Alcoholism[tiab]** |
| [#22](https://www.ncbi.nlm.nih.gov/pubmed/advanced) | Search **Alcohol[tiab]** |
| [#21](https://www.ncbi.nlm.nih.gov/pubmed/advanced) | Search **Alcoholic Beverages[Mesh]** |
| [#20](https://www.ncbi.nlm.nih.gov/pubmed/advanced) | Search **(#14 OR #19)** |
| [#19](https://www.ncbi.nlm.nih.gov/pubmed/advanced) | Search **(#17 AND #18)** |
| [#18](https://www.ncbi.nlm.nih.gov/pubmed/advanced) | Search **Model*[tiab]** |
| [#17](https://www.ncbi.nlm.nih.gov/pubmed/advanced) | Search **(#15 OR #16)** |
| [#16](https://www.ncbi.nlm.nih.gov/pubmed/advanced) | Search **Attributable*[tiab]** |
| [#15](https://www.ncbi.nlm.nih.gov/pubmed/advanced) | Search **Burden[tiab]** |
| [#14](https://www.ncbi.nlm.nih.gov/pubmed/advanced) | Search **(#1 OR #2 OR #3 OR #4 OR #5 OR #6 OR #7 OR #8 OR #9 OR #10 OR #11 OR #12 OR #13)** |
| [#13](https://www.ncbi.nlm.nih.gov/pubmed/advanced) | Search **Modelling Methodol*[tiab]** |
| [#12](https://www.ncbi.nlm.nih.gov/pubmed/advanced) | Search **Public Health Intervent*[tiab]** |
| [#11](https://www.ncbi.nlm.nih.gov/pubmed/advanced) | Search **Public Health Model[tiab]** |
| [#10](https://www.ncbi.nlm.nih.gov/pubmed/advanced) | Search **Policy Model[tiab]** |
| [#9](https://www.ncbi.nlm.nih.gov/pubmed/advanced) | Search **QALY*[tiab]** |
| [#8](https://www.ncbi.nlm.nih.gov/pubmed/advanced) | Search **DALY*[tiab]** |
| [#7](https://www.ncbi.nlm.nih.gov/pubmed/advanced) | Search **Risk Assess*[tiab]** |
| [#6](https://www.ncbi.nlm.nih.gov/pubmed/advanced) | Search **Comparative Risk[tiab]** |
| [#5](https://www.ncbi.nlm.nih.gov/pubmed/advanced) | Search **Cost-Benefit Analysis[Mesh]** |
| [#4](https://www.ncbi.nlm.nih.gov/pubmed/advanced) | Search **Decision Analys*[tiab]** |
| [#3](https://www.ncbi.nlm.nih.gov/pubmed/advanced) | Search **Econometric Model*[tiab]** |
| [#2](https://www.ncbi.nlm.nih.gov/pubmed/advanced) | Search **Economic Model*[tiab]** |
| [#1](https://www.ncbi.nlm.nih.gov/pubmed/advanced) | Search **Models, Economic[Mesh]** |

Cochrane Library (Wiley) 22-06-2017

ID Search Hits

#1 MeSH descriptor: [Models, Economic] explode all trees 2017

#2 Economic Model*:ti,ab,kw (Word variations have been searched)

#3 Econometric Model*:ti,ab,kw (Word variations have been searched)

#4 Decision Analys*:ti,ab,kw (Word variations have been searched)

#5 MeSH descriptor: [Cost-Benefit Analysis] explode all trees

#6 Comparative Risk:ti,ab,kw (Word variations have been searched)

#7 Risk Assess*:ti,ab,kw (Word variations have been searched)

#8 DALY*:ti,ab,kw (Word variations have been searched)

#9 QALY*:ti,ab,kw (Word variations have been searched)

#10 Policy Model:ti,ab,kw (Word variations have been searched)

#11 Public Health Model:ti,ab,kw (Word variations have been searched)

#12 Public Health Intervent*:ti,ab,kw (Word variations have been searched)

#13 Modelling Methodol*:ti,ab,kw (Word variations have been searched)

#14 #1 or #2 or #3 or #4 or #5 or #6 or #7 or #8 or #9 or #10 or #11 or #12 or #13

#15 Burden:ti,ab,kw (Word variations have been searched)

#16 Attributable*:ti,ab,kw (Word variations have been searched)

#17 #15 or #16

#18 Model*:ti,ab,kw (Word variations have been searched)

#19 #17 and #18

#20 #14 or #19

#21 MeSH descriptor: [Alcoholic Beverages] explode all trees

#22 Alcohol:ti,ab,kw (Word variations have been searched)

#23 Alcoholism:ti,ab,kw (Word variations have been searched)

#24 Alcoholic*:ti,ab,kw (Word variations have been searched)

#25 Drinker*:ti,ab,kw (Word variations have been searched)

#26 #21 or #22 or #23 or #24 or #25

#27 #20 and #26

PsycINFO (EBSCO) 29-06-2017

| # | Query |
| --- | --- |
| S23 | S16 AND S22 |
| S22 | S17 OR S18 OR S19 OR S20 OR S21 |
| S21 | TI Drinker* OR AB Drinker* |
| S20 | TI Alcoholic* OR AB Alcoholic* |
| S19 | TI Alcoholism OR AB Alcoholism |
| S18 | TI Alcohol OR AB Alcohol |
| S17 | DE "Alcoholic Beverages" OR DE "Beer" OR DE "Liquor" OR DE "Wine" |
| S16 | S10 AND S15 |
| S15 | S11 OR S12 OR S13 OR S14 |
| S14 | TI Public Health Model OR AB Public Health Model |
| S13 | TI Policy Model OR AB Policy Model |
| S12 | TI Modelling Methodol* OR AB Modelling Methodol* |
| S11 | TI Economic Model* OR AB Economic Model* |
| S10 | S1 OR S2 OR S3 OR S4 OR S5 OR S6 OR S7 OR S8 OR S9 |
| S9 | TI Attributable* OR AB Attributable* |
| S8 | TI Burden OR AB Burden |
| S7 | TI Public Health Intervent* OR AB Public Health Intervent* |
| S6 | TI QALY* OR AB QALY* |
| S5 | TI DALY* OR AB DALY* |
| S4 | TI Risk Assess* OR AB Risk Assess* |
| S3 | TI Comparative Risk OR AB Comparative Risk |
| S2 | TI Cost-Benefit* OR AB Cost-Benefit* |
| S1 | TI Decision Analy* OR AB Decision Analy* |

EconLit (EBSCO) 4-07-2017

| # | Query |
| --- | --- |
| S25 | S19 AND S24 |
| S24 | S20 OR S21 OR S22 OR S23 |
| S23 | TI Drinker* OR AB Drinker* |
| S22 | TI Alcoholic* OR AB Alcoholic* |
| S21 | TI Alcoholism OR AB Alcoholism |
| S20 | TI Alcohol OR AB Alcohol |
| S19 | S13 OR S18 |
| S18 | S16 AND S17 |
| S17 | TI Model* OR AB Model* |
| S16 | S14 OR S15 |
| S15 | TI Attributable* OR AB Attributable* |
| S14 | TI Burden OR AB Burden |
| S13 | S1 OR S2 OR S3 OR S4 OR S5 OR S6 OR S7 OR S8 OR S9 OR S10 OR S11 OR S12 |
| S12 | TI Modelling Methodol* OR AB Modelling Methodol* |
| S11 | TI Public Health Intervent* OR AB Public Health Intervent* |
| S10 | TI Public Health Model OR AB Public Health Model |
| S9 | TI Policy Model OR AB Policy Model |
| S8 | TI QALY* OR AB QALY* |
| S7 | TI DALY* OR AB DALY* |
| S6 | TI Risk Assess* OR AB Risk Assess* |
| S5 | TI Comparative Risk OR AB Comparative Risk |
| S4 | TI Cost-Benefit OR AB Cost-Benefit |
| S3 | TI Decision Analy* OR AB Decision Analy* |
| S2 | TI Econometric Model* OR AB Econometric Model* |
| S1 | TI Economic Model* OR AB Economic Model* |

LILACS (BVS) 4-07-2017

(MH Modelos Económicos OR MH Análisis Costo-Beneficio OR (Econom$ AND Model$) OR ((Decision OR Decisão) AND Analis$)) OR ((Comparati$ OR Assess$ Evalua$) AND (Risk OR Riesgo$ OR Risco$)) OR DALY$ OR QALY$ OR ((Model$ OR Interven$) AND (Policy OR Public$))) AND (((Burden OR Carga OR Attributable OR Atribui$) AND (Model$)) AND (MH Bebidas Alcohólicas OR Alcohol$ OR Drinker$ OR Bebedor$) [Palabras]

EMBase (Elsevier) 4-07-2017

| #27 | #20 AND #26 |
| --- | --- |
| #26 | #21 OR #22 OR #23 OR #24 OR #25 |
| #25 | drinker*:ti,ab |
| #24 | alcoholic*:ti,ab |
| #23 | alcoholism:ti,ab |
| #22 | alcohol:ti,ab |
| #21 | 'alcoholic beverage'/exp |
| #20 | #14 OR #19 |
| #19 | #17 AND #18 |
| #18 | model*:ti,ab |
| #17 | #15 OR #16 |
| #16 | attributable*:ti,ab |
| #15 | burden:ti,ab |
| #14 | #1 OR #2 OR #3 OR #4 OR #5 OR #6 OR #7 OR #8 OR #9 OR #10 OR #11 OR #12 OR #13 |
| #13 | 'modelling methodology':ti,ab OR 'modelling methodologies':ti,ab |
| #12 | 'public health model':ti,ab OR 'public health models':ti,ab |
| #11 | 'public health intervention':ti,ab OR 'public health interventions':ti,ab |
| #10 | 'public health model':ti,ab |
| #9 | 'policy model':ti,ab |
| #8 | qaly*:ti,ab |
| #7 | daly*:ti,ab |
| #6 | 'risk assessment':ti,ab |
| #5 | 'comparative risk':ti,ab |
| #4 | 'cost benefit analysis'/exp |
| #3 | 'decision analysis':ti,ab |
| #2 | 'economic model':ti,ab OR 'economic models':ti,ab |
| #1 | 'economic model'/exp |

# **Appendix 2** Checklist for model evaluation, and score frame

# Checklist

| **Checklist item** | **Score obtained (Yes = 1, No = 0)** |
| --- | --- |
| **Strength** | |
| **Objective/purpose:**  1. Whether there is clear statement about the questions that the model aimed to answer |  |
|  |  |
| **Model formulation and transparency:**  Whether there is clear description about   1. model structure or model statement (including formula) 2. model specification 3. model assumptions 4. model derivation 5. variable used in the model (response, predictors, potential confounders, lag time) 6. method of parameter estimation and inference 7. model building (variable selection) process 8. model diagnostic and adequacy checking 9. theoretical ground of the model |  |
|  |  |
| **Data:**  Whether there is clear description about   1. source of data 2. method of data collection (study design) 3. sample size and determination process 4. method of assessment (data measurement) |  |
| **Model findings:**  Whether following information are available   1. necessary estimates (prevalence/burden measure/incidence) 2. standard errors and 95% confidence intervals of the estimates 3. model fit information 4. compliance of the model findings with real scenario |  |
| **Model validation:**  1. Whether model validation was performed |  |
| **Limitation** | |
| **Sensitivity analysis:**  1. Whether sensitivity analyses was carried out |  |
| **Addressing missing data**  1. Whether there is explanation about how missing data were addressed |  |
| **Dissemination and expert involvement:**   1. Whether model was disseminated prior to final development of model 2. Whether expert opinion were incorporated to develop the model |  |
| **Policymakers involvement:**   1. Whether policymakers were involved in the model development 2. Whether any policy was recommended on the basis of model derived findings |  |
| **Limitation discussed in the paper:**  Whether potential methodological limitations of the model were discussed |  |

| **Reproducibility** | |
| --- | --- |
| **Reproducibility:**   1. Whether model is available and accessible to user 2. Whether data are available 3. Whether codes are available 4. Whether user manual to develop the model is available |  |

# Scoring framework

| **Criteria** | **Item** | **Score assigned** | **Score obtained** |
| --- | --- | --- | --- |
| Strength | Objectives of the model | 1 |  |
|  | Model formulation and transparency | 9 |  |
|  | Data | 4 |  |
|  | Model findings | 4 |  |
|  | Model validation | 1 |  |
| Limitation | Sensitivity analysis | 1 |  |
|  | Addressing missing data | 1 |  |
|  | Dissemination and expert involvement | 2 |  |
|  | Policymakers involvement | 2 |  |
|  | Limitation discussed in the paper | 1 |  |
| Reproducibility | Reproducibility of the model | 4 |  |
| Total | | 30 |  |
